# NSs Virulence Factor of Rift Valley Fever Virus Engages the F-Box Proteins FBXW11 and β-TRCP1 To Degrade the Antiviral Protein Kinase PKR

**DOI:** 10.1128/JVI.00016-16

**Published:** 2016-06-10

**Authors:** Markus Kainulainen, Simone Lau, Charles E. Samuel, Veit Hornung, Friedemann Weber

**Affiliations:** a Institute for Virology, Philipps-Universität Marburg, Marburg, Germany; b Institute for Virology, FB10-Veterinary Medicine, Justus-Liebig-Universität Gießen, Giessen, Germany; c Department of Molecular, Cellular and Developmental Biology, University of California, Santa Barbara, California, USA; d Biomolecular Sciences and Engineering Program, University of California, Santa Barbara, California, USA; e Institute of Molecular Medicine, University Hospital, Universität Bonn, Bonn, Germany; f Gene Center and Department of Biochemistry, Ludwig-Maximilians-Universität München, Munich, Germany; St Jude Children's Research Hospital

## Abstract

Rift Valley fever virus (RVFV, family Bunyaviridae, genus Phlebovirus) is a relevant pathogen of both humans and livestock in Africa. The nonstructural protein NSs is a major virulence factor known to suppress the type I interferon (IFN) response by inhibiting host cell transcription and by proteasomal degradation of a major antiviral IFN effector, the translation-inhibiting protein kinase PKR. Here, we identified components of the modular SCF (Skp1, Cul1, F-box protein)-type E3 ubiquitin ligases as mediators of PKR destruction by NSs. Small interfering RNAs (siRNAs) against the conserved SCF subunit Skp1 protected PKR from NSs-mediated degradation. Consequently, RVFV replication was severely reduced in Skp1-depleted cells when PKR was present. SCF complexes have a variable F-box protein subunit that determines substrate specificity for ubiquitination. We performed an siRNA screen for all (about 70) human F-box proteins and found FBXW11 to be involved in PKR degradation. The partial stabilization of PKR by FBXW11 depletion upregulated PKR autophosphorylation and phosphorylation of the PKR substrate eIF2α and caused a shutoff of host cell protein synthesis in RVFV-infected cells. To maximally protect PKR from the action of NSs, knockdown of structurally and functionally related FBXW1 (also known as β-TRCP1), in addition to FBXW11 deletion, was necessary. Consequently, NSs was found to interact with both FBXW11 and β-TRCP1. Thus, NSs eliminates the antiviral kinase PKR by recruitment of SCF-type E3 ubiquitin ligases containing FBXW11 and β-TRCP1 as substrate recognition subunits. This antagonism of PKR by NSs is essential for efficient RVFV replication in mammalian cells.

**IMPORTANCE** Rift Valley fever virus is a pathogen of humans and animals that has the potential to spread from Africa and the Arabian Peninsula to other regions. A major virulence mechanism is the proteasomal degradation of the antiviral kinase PKR by the viral protein NSs. Here, we demonstrate that NSs requires E3 ubiquitin ligase complexes of the SCF (Skp1, Cul1, F-box protein) type to destroy PKR. SCF-type complexes can engage variant ubiquitination substrate recognition subunits, and we found the F-box proteins FBXW11 and β-TRCP1 to be relevant for the action of NSs against PKR. Thus, we identified the host cell factors that are critically needed by Rift Valley fever virus to uphold its replication against the potent antiviral kinase PKR.

## INTRODUCTION

Rift Valley fever virus (RVFV, family Bunyaviridae, genus Phlebovirus) is a significant mosquito-transmitted pathogen (1, 2). It causes large and devastating epidemics in Africa and the Arabian Peninsula, typically killing hundreds of humans and thousands of farm animals (3). The recurrent outbreaks in ruminants are characterized by so-called “abortion storms” and the deaths of up to 100% of newborn animals. In adult animals, the mortality rate due to hepatitis or hemorrhage typically ranges from 10 to 30%, and among hospitalized humans patients, it is 10 to 20%. RVFV is able to use a wide variety of mosquito species as vectors (4). This feature, along with the severity of the disease and the impressive epidemic potential, has raised serious concerns about the introduction of RVFV into the European Union or the United States, as a high number of susceptible animals and competent vectors are present in both regions (5, 6).

RVFV has a negative-sense RNA genome divided into three segments designated L, M, and S. The segments encode a basic set of proteins, namely, the RNA polymerase (RNAP) L (L segment), the glycoproteins Gn and Gc (M segment), and the nucleocapsid protein N (S segment). Moreover, RVFV encodes nonstructural proteins (NSm proteins, a 78-kDa protein, and NSs) that interfere with apoptosis, the cell cycle, insect permissiveness, and innate host defenses (7–12).

The NSs protein is the main virulence factor of RVFV; it acts by suppressing the mammalian antiviral type I interferon (IFN) system at multiple levels. Type I IFNs (IFN-α/β) are cytokines that are transcriptionally upregulated in virus-infected cells (13). Secreted IFNs act through their cognate cellular receptor to establish an antiviral state by the activation of the so-called IFN-stimulated genes (ISGs) (13, 14). Several protein products of ISGs possess antiviral activity. Among them is PKR, a potent inhibitor of protein synthesis (15, 16). RVFV NSs was initially described as an efficient inhibitor of IFN induction (17) that interferes with host cell RNAP II function (18). NSs acts through recruitment of the transcriptional repressor SAP30 to the IFN promoter (19) and the impairment of general transcription factor TFIIH through sequestration of the p44 subunit and proteasomal destruction of the p62 subunit (20–23). We and others found that NSs also counteracts the IFN response by targeting the IFN effector PKR (9, 10). NSs triggers the proteasomal degradation of PKR, a protein that otherwise would become activated by viral RNA and block RVFV replication (9).

To begin to elucidate the molecular mechanisms of the action of RVFV NSs, we had carried out a large-scale interactome analysis by using tandem affinity purification and one-dimensional gel liquid chromatography-mass spectrometry (24). One of the host cell interactors identified turned out to be a ubiquitin E3 ligase, FBXO3, that was essential for the proteasomal degradation of the TFIIH subunit p62 by NSs (22). FBXO3 interaction with NSs was responsible for the proteasomal destruction of TFIIH-p62 but not the degradation of the antiviral kinase PKR (22). Therefore, the degradation of PKR by NSs is expected to be mediated by another host cell factor. Here, we describe the identification of two other E3 ubiquitin ligases, the F-box proteins FBXW11 and β-TRCP1, that are recruited by NSs to destroy PKR in RVFV-infected cells.

## MATERIALS AND METHODS

### Cells, viruses, and reagents.

Human HeLa and A549 cells, primate Vero E6 cells, and hamster BSR-T7/5 cells were cultivated in Dulbecco's modified Eagle's medium supplemented with 5% (HeLa) or 10% (A549, Vero E6, BSR-T7/5) fetal calf serum (FCS), 4 mM (HeLa) or 2 mM (A549, Vero E6, and BSR-T7/5) l-glutamine, 50 U/ml penicillin, and 50 μg/ml streptomycin. Information on the use of the different cell lines is provided in the figure legends. RVFV strains rZH548 (recombinant wild type [WT]) and clone 13 (an NSs-deficient mutant strain) were propagated on Vero E6 cells under biosafety level 3 conditions as described previously (22).

### Virus titration.

Virus-containing cell culture supernatant titers were determined on BSR-T7/5 cells. Near-confluent monolayers in 96-well plates were infected with dilution series of the samples for 1 h. After removal of the inocula, cells were overlaid with minimum essential medium-based medium containing 2% FCS, penicillin-streptomycin, 0.01% DEAE-dextran (Sigma-Aldrich), and 1.5% Avicel (FMC Biopolymers). After 24 h, the plates were fixed with 4% paraformaldehyde, permeabilized by four washes with 0.1% Triton X-100 in phosphate-buffered saline (PBS), and blocked with 5% nonfat dry milk in 0.1% Tween 20–Tris-buffered saline (TBS-T). RVFV-infected cells were immunostained with a mouse polyclonal antiserum raised against RVFV clone 13 (9) as the primary antibody and IRDye800-conjugated goat anti-mouse IgG polyclonal antibody (Rockland) as the secondary antibody. The DNA-binding stain DRAQ5 (eBioscience) was used to stain the cell monolayer. Fluorescent foci were imaged with the Odyssey instrument (LI-COR).

### Plasmid constructs.

Total cell RNA was used for reverse transcription (RT) with random hexamer primers and PCR with specific primers. cDNAs for Skp1, FBXW11, and β-TRCP1 were derived from A549 cells, whereas 3×FLAG-tagged RVFV NSs originated from WT RVFV strain ZH548. The cDNA amplicons were T/A cloned into pcDNA3.1 (Invitrogen). pcDNA3.1 3×HA-ΔMx was cloned by ligating the PCR-amplified insert of pI.18/3×FLAG_ΔMx (25) via the BamHI and XhoI sites. All inserts were sequenced and verified to match the corresponding GenBank entries (NM_006930.3 for Skp1, NM_033645.2 for FBXW11, NM_033637.3 for β-TRCP1, and DQ380151.1 for RVFV NSs). Detailed cloning strategies and primers are available upon request.

### Western blot analyses.

Cells were washed with PBS and lysed with a 1:1 mixture of tissue protein extraction reagent (Thermo Scientific) with added phosphatase and protease inhibitors and 2× sample buffer (62.5 mM Tris from a 0.5 M stock [pH 6.8], 25% glycerol, 2% SDS, 0.01% bromophenol blue, 5% 2-mercaptoethanol). The samples were boiled twice for 10 min and then separated by SDS-PAGE, blotted onto polyvinylidene difluoride membranes, and blocked with 5% (wt/vol) nonfat dry milk powder in TBS-T. The membranes were probed with primary antibodies against the following targets: hemagglutinin (HA) tag (Abcam rabbit anti-HA polyclonal antibody ab9110; 1:4,000), TFIIH-p62 (Abcam mouse monoclonal antibody anti-GTF2H1 ab5519; 1:1,000), Skp1 (Abcam mouse monoclonal antibody EPR3304; 1:1,000), FLAG tag (Sigma rabbit anti-FLAG polyclonal antibody F7425; 1:3,200), β-actin (Cell Signaling mouse monoclonal antibody 8H10D10; 1:2,000), eIF2-α (Cell Signaling mouse monoclonal antibody 2103; 1:2,000), phospho-S52 eIF2-α (Invitrogen/Biosource rabbit polyclonal antibody 44728G; 1:2,000), anti-puromycin (Kerafast mouse monoclonal antibody 3RH11; 1:1,000), PKR (mouse monoclonal antibody 71/10; 1:1,000) (26), phospho-Thr446 PKR (Epitomics rabbit monoclonal antibody 1120-1; 1:2,000), and a mouse anti-RVFV N polyclonal antibody (1:2,000) (9). Peroxidase-conjugated anti-mouse and anti-rabbit IgG polyclonal antibodies (Thermo Fisher) were used as secondary antibodies at a dilution of 1:40,000.

### siRNA screen targeting F-box proteins.

A small interfering RNA (siRNA) screen with infection and quantification of the total PKR immunofluorescence signal in the cell monolayer (In-Cell Western assay principle; LI-COR) was established to determine whether PKR degradation in RVFV-infected cells depends on an F-box protein.

Genes for F-box protein targets were knocked down with siRNA pools of Human siGENOME siRNA Library—Ubiquitin Conjugation Subset 2 with siGENOME Non-Targeting siRNA Pool 2 (Dharmacon) as the negative control. Transfection complexes with RNAiMAX reagent (Invitrogen) were prepared in 96-well plates according to the manufacturer's instructions. A549 cells were seeded on top of the complexes so that a final concentration of 50 nM total siRNA was obtained. The cells were allowed to attach for 4 to 6 h, and the medium was changed. Three days later, the cells were again transfected with 50 nM total siRNA, this time by adding the transfection complexes to the attached cells. The medium was changed 4 to 6 h after transfection. One day later, the cells were infected with recombinant WT RVFV strain rZH548 at a multiplicity of infection (MOI) of 10 or mock infected. Seven hours later, the plates were fixed with 4% paraformaldehyde in PBS. Cells were permeabilized by four washes with 0.1% Triton X-100 in PBS, washed with PBS and TBS, and then blocked with 5% nonfat dry milk in TBS-T (blocking buffer). Staining for PKR was then done with antibody clone YE350 (Epitomics) in the blocking buffer at +4°C overnight (for background determination, some wells were mock stained). Secondary antibody staining was done with IRDye800-conjugated goat anti-rabbit IgG polyclonal antibody (Rockland) in blocking buffer. Nonspecific cell stain Sapphire700 (LI-COR) and DNA-binding stain DRAQ5 (eBioscience) were used according to the instructions of LI-COR to quantify well-to-well variation in cell amounts. For background signal determination, wells that were not stained with the primary antibody were stained with the secondary antibody in the absence of the normalization dyes.

Raw signals measured with the Odyssey instrument (LI-COR) were corrected for the background by reducing signals from wells without the primary antibody, Sapphire700, or DRAQ5. The corrected PKR signal was then normalized to the corrected cell stain signal, and the signal from the mock-infected, control siRNA-treated well was set as 1 to represent the total PKR under control conditions. Other signals are expressed relative to this value.

### siRNA transfections.

Cells were reverse transfected two times with 50 nM siRNA mixtures with Lipofectamine RNAiMAX (Invitrogen) as described previously (22). AllStars Negative Control siRNA and validated pools of four siRNAs against mRNAs for FBXO3 (Qiagen GeneSolution GS26273), Skp1 (Qiagen GeneSolution GS6500), FBXW11 (Qiagen GeneSolution GS23291), and β-TRCP1 (Dharmacon D-003463-01 to -04) were used.

### Real-time RT-PCR.

An aliquot of 100 ng of total RNA was isolated from cells, reverse transcribed, and analyzed by SYBR green-based PCR as described previously (22). mRNA levels of human FBXW11, β-TRCP1, and glyceraldehyde 3-phosphate dehydrogenase (used for normalization) were determined with QuantiTect primers (Qiagen) QT00083251, QT00080787, and QT01192646, respectively.

### Puromycin labeling.

Protein synthesis of cells can be determined by puromycin labeling of nascent protein chains (27). To measure ongoing translation during RVFV infection, cells were incubated with medium containing 1 μM puromycin (Sigma P7255-100MG) for 30 min before the cells were processed for Western blot analysis. The labeling did not influence PKR, p-PKR, eIF2α, p-eIF2α, RVFV N, or β-actin levels compared to those in mock-labeled samples (data not shown).

### Generation of FBXW11 knockout cell lines.

A critical exon of the FBXW11gene was targeted with CRISPR/Cas9 at the following target site: 5′-GTGGACGACACAACTTGCAGAGG-3′. A549 cells were transfected with pRZ-mCherry-Cas9 and the single guide RNA construct at a ratio of 150 ng to 50 ng per well as previously described (28). Two days later, targeted cells were plated for limiting dilution cloning, and 2 weeks later, the monoclones thus obtained were selected by bright-field microscopy. The genotype of these clones was determined by deep sequencing on an Illumina MiSeq with the following primer sequences: FBXW11_fwd, **ACA CTC TTT CCC TAC ACG ACG** CTC TTC CGA TCT ***AGC AAC GCC AAA GTC TGA AAC CT***; FBXW11_rev, **TGA CTG GAG TTC AGA CGT GTG** CTC TTC CGA TCT ***GTC TAA CAC ATA GCC ACG ATT CT*** (Illumina adapter sequences are in bold roman type, linker sequences are underlined, and target region-specific sequences are in bold italic type). Data were analyzed with OutKnocker.org (29), and clones with all-allelic frameshift mutations were considered for further experiments. One clone (termed H8) that contained a uniform 4-bp deletion in the target sequence (5′-GTGGACGACACAAC----AGAGG-3′) was selected as the FBXW11 knockout cell line. Key data obtained with these cells were reproduced with an independent FBXW11 knockout cell clone.

### Coimmunoprecipitation assays.

3×HA-tagged Skp1, FBXW11, β-TRCP1, ΔMx, and 3×FLAG-tagged RVFV NSs protein were produced individually by *in vitro* transcription/translation with a T7 TnT Quick Coupled system (Promega) in accordance with the manufacturer's specifications. The *in vitro*-expressed proteins with the HA tag were coupled to Dynabeads (Invitrogen) with anti-HA.11 antibody (Covance). After three washing steps with a buffer containing 10 mM piperazine-*N*,*N*′-bis(2-ethanesulfonic acid) (pH 6.8), 100 mM NaCl, 300 mM sucrose, 3 mM CaCl_2_, 1 mM EDTA, 1 mM dithiothreitol, 1 mM phenylmethylsulfonyl fluoride, protease inhibitor cocktail (Roche), and 0.5% Triton X-100, the beads were mixed with equal amounts of *in* *vitro*-translated 3×FLAG-RVFV NSs and incubated for 2 h at room temperature in the same buffer. After three further washings with the same buffer, the immunocomplexes were eluted by boiling the beads in SDS-loading buffer. One-tenth of the input reaction mixture, as well as the complete immunoprecipitate, was analyzed by Western blotting.

## RESULTS

### The F-box protein interactor Skp1 is involved in PKR antagonism by RVFV.

**FIG 1:**
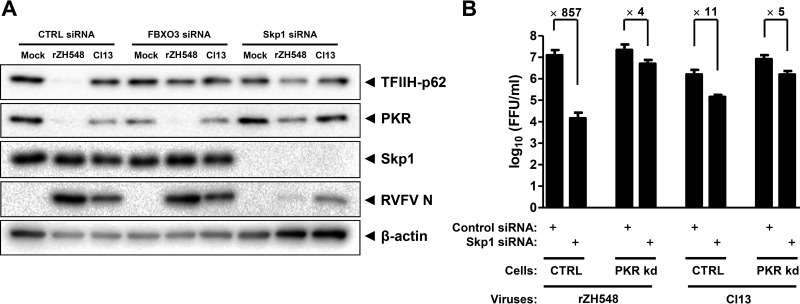
Skp1 is required for the degradation of TFIIH-p62 and PKR by RVFV NSs. (A) Effect of Skp1 knockdown on the degradation activity of RVFV NSs. HeLa cells were transfected with siRNA against FBXO3 or Skp1 mRNA and then infected with WT RVFV (rZH548) or clone 13 (Cl13) at an MOI of 10. A nontarget siRNA (CTRL) and uninfected (mock) cells were used as negative controls for knockdown and infection effects, respectively. At 6 h p.i., lysates were analyzed by Western blotting for the presence of the proteins indicated. (B) NSs-, PKR-, and Skp1-dependent growth of RVFV. HeLa cells with stable knockdown (kd) of PKR and matching control HeLa cells (31) were treated with a siRNA mixture against Skp1 mRNA or with a control siRNA. The cells were then infected with WT RVFV (rZH548) or clone 13 at an MOI of 1. Virus titers were determined at 24 h p.i. Three replicate wells were infected per experiment, and mean values were calculated. The experiment was repeated four times, and mean values and standard deviations of the four independent repeats, as well as fold differences between siRNA treatments, are shown.

Skp1 is an interactor and cofactor of F-box proteins (30) that, together with FBXO3, enables NSs to degrade TFIIH-p62 (22). During our investigations of FBXO3, we found that removal of Skp1 had a much broader effect on the RVFV phenotype than removal of FBXO3. Figure 1 shows the results of such an experiment. Cells treated with the negative-control siRNA displayed the expected loss of TFIIH-p62 and PKR when infected with NSs-expressing recombinant WT RVFV (rZH548), whereas infection with the NSs-deficient RVFV mutant strain clone 13 (Cl13) spared both proteins (Fig. 1A). When cells were pretreated with an siRNA against FBXO3 and infected with WT RVFV, degradation of TFIIH-p62 was rescued in a specific manner. In cells pretreated with a Skp1 siRNA, in contrast, both TFIIH-p62 and PKR were rescued from the action of NSs.

Skp1 knockdown cells also exhibited reduced levels of viral N, indicating impairment of RVFV replication. This could occur for two different reasons. One possibility is that Skp1 is generally required for viral replication. Another possibility is that Skp1 is specifically involved in NSs-mediated PKR degradation. Since PKR is an inhibitor of RVFV (9), protection of PKR from degradation by a lack of Skp1 would affect viral multiplication. To distinguish between these alternatives, we employed cells deficient in PKR expression because of stable transfection with a short hairpin RNA (shRNA) plasmid (31). Infection of control shRNA-transfected cells (i.e., those expressing PKR) confirmed the requirement of Skp1 for the growth of WT RVFV, as Skp1 knockdown by siRNA treatment lowered titers by almost 3 orders of magnitude (Fig. 1B). In PKR-deficient cells, however, the virus titer difference between control siRNA and Skp1 siRNA was reduced to less than 1 order of magnitude. Moreover, when the same set of experiments was performed in parallel with NSs-deficient RVFV mutant clone 13, Skp1 knockdown made only a minor difference, independent of whether PKR-deficient or PKR-sufficient cells were used. In agreement with our previous findings (9), the replication of clone 13 was impaired in the presence of PKR. Thus, taking the results together, both PKR and NSs are required for the major inhibitory effect of Skp1 depletion on RVFV replication, indicating that Skp1 is specifically involved in the anti-PKR activity of NSs-expressing WT RVFV.

### Human F-box protein screening.

**FIG 2:**
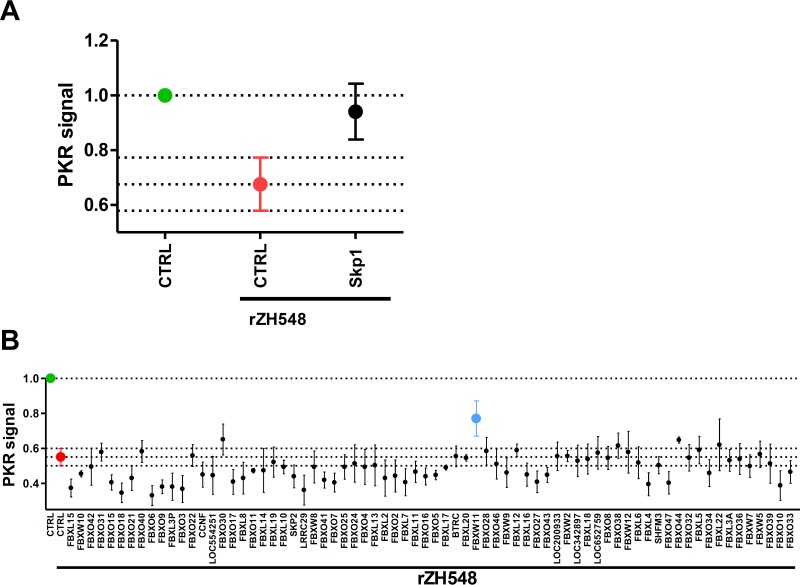
siRNA screen for F-box proteins involved in PKR degradation by NSs. Human A549 cells seeded into a 96-well plate were transfected with siRNA against the gene for the conserved SCF complex component Skp1 (A) or against all human F-box proteins (B) and infected with recombinant WT RVFV (rZH548) at an MOI of 10 or mock infected. At 7 h p.i., cells were fixed, permeabilized, and stained with an antibody to PKR. Stains binding DNA and cells in a nonspecific manner were additionally used to normalize for well-to-well variations. The background-corrected signal from the mock-infected, control (CTRL) siRNA-treated well was arbitrarily set as 1 to represent total PKR under control conditions (green spot). Other signals are expressed relative to this value. The red spot indicates the signal for PKR degradation by RVFV NSs, and the blue spot in panel B highlights FBXW11 as the most significant hit. The experiment was repeated three times, and average values and standard deviations are shown.

Skp1 is an essential component of the modular SCF (Skp1, Cul1, F-box protein [and Rbx1]) E3 ubiquitin ligases. It forms the bridge between the E3 core machinery that is common to all SCF ubiquitin ligases and the variable F-box protein component that selects the substrates of ubiquitination (30). The requirement of Skp1 for NSs-mediated PKR degradation indicates the involvement of an unknown F-box protein. The human genome encodes 69 different F-box proteins and some putative candidate open reading frames (32). We designed an siRNA screening assay based on the quantification of intracellular PKR levels in infected cells. Cells were seeded into 96-well plates, and each well was incubated with an siRNA pool against a particular candidate F-box protein and infected with WT RVFV. Cells were then fixed and stained for PKR with primary and fluorescent secondary antibodies, and the fluorescence signal was quantified and normalized to the cell number. To test the feasibility of the screening procedure, we first employed an siRNA knockdown of Skp1 (Fig. 2A). In cells transfected with a negative-control siRNA, the baseline PKR signal (green spot) dropped considerably upon infection with WT RVFV (red spot), as expected. This demonstrates that PKR degradation by NSs can be monitored by such a method. The siRNA pools against Skp1 rescued levels of PKR, confirming the involvement of an SCF-type ubiquitin ligase in PKR degradation by RVFV. Screening with the different siRNAs pools (Fig. 2B) showed that most F-box proteins had no major influence on PKR destruction. The best hit of the screening was the knockdown of F-box protein FBXW11 (also called β-TRCP2), which clearly and reproducibly rescued PKR levels (blue spot). Of note, rescue of PKR by none of the siRNA pools achieved statistical significance when appropriate statistical testing for multiple comparisons was applied. Nonetheless, the FBXW11 siRNA pool consistently resulted in the highest retained PKR levels. The contribution of FBXW11 to NSs-induced PKR degradation was therefore evaluated in further experiments.

### FBXW11 contributes to PKR degradation by NSs.

The FBXW11 siRNA experiment was repeated in a larger setting that included NSs-mutated RVFV strain clone 13 as a negative control and Western blot analyses for several cellular and viral proteins. Besides PKR, we analyzed the other target of NSs, TFIIH-p62, as well as phospho-PKR and phospho-eIF2-α as markers of PKR activation, and RVFV N, eIF2-α, and β-actin as markers of infection and protein loading, respectively. It is known that activation by viral RNA results in PKR autophosphorylation and the subsequent phosphorylation of eIF2-α, one of its major substrates (15). Phosphorylated eIF2-α is a potent inhibitor of translation, resulting in the shutoff of protein synthesis. Therefore, we also analyzed *de novo* protein synthesis by the nonradioactive puromycin labeling method in a time window of 30 min before cell lysis (27).

**FIG 3:**
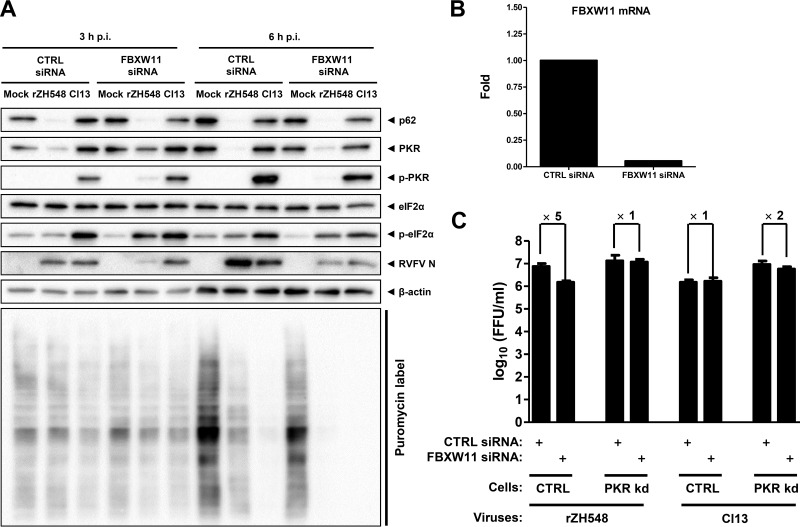
FBXW11 is involved in PKR degradation by NSs. (A) FBXW11 knockdown and PKR degradation in infected cells. A549 cells were transfected with siRNAs against FBXW11 mRNA and then infected with WT RVFV (rZH548) or clone 13 (Cl13) at an MOI of 10 for 3 or 6 h, as indicated. Nascent protein chains were labeled by incubating cells with puromycin for 30 min before lysis. Samples were analyzed by Western blotting for the presence of the proteins indicated and for the puromycin label. (B) Efficiency of FBXW11 siRNA knockdown (kd), shown by RT-quantitative PCR. (C) NSs-, PKR-, and FBXW11-dependent growth of RVFV. PKR-sufficient and PKR-deficient HeLa cells were treated with siRNAs against the FBXW11 mRNA or with a control (CTRL) siRNA and infected with rZH548 or clone 13 at an MOI of 1, and virus titers were determined at 24 h p.i. Three replicate wells were infected per experiment, and mean values were calculated. The experiment was repeated four times, and mean values and standard deviations of the four independent repeats, as well as fold differences between siRNA treatments, are shown.

Figure 3A shows that, as expected, infection of control siRNA-transfected cells with NSs-mutated clone 13 activated the phosphorylation of both PKR and eIF2-α and triggered a translational shutoff that was apparent at 6 h postinfection (p.i.). NSs-expressing WT RVFV, as expected, did not activate the phosphorylation of PKR or eIF2-α because it destroyed PKR. Therefore, it allowed ongoing protein synthesis, albeit at a lower rate than in uninfected cells. Most likely, this reduction is due to the general host cell shutoff by NSs-mediated RNAP II inhibition. As observed in the siRNA screening described above, removal of FBXW11 led to increased PKR levels in WT RVFV-infected cells. Selectivity for PKR was demonstrated by the fact that TFIIH-p62 was still entirely destroyed in WT RVFV-infected FBXW11 siRNA cells. However, PKR rescue was only seen at 3 h p.i., whereas at the longer infection time, the PKR signal was diminished (albeit not eliminated, as with the control siRNA) by WT RVFV. The partial rescue of PKR levels in FBXW11-depleted cells permitted the virus-induced phosphorylation of PKR slightly and that of eIF2-α strongly and resulted in a shutoff of protein synthesis and reduction of virus replication. The efficiency of this siRNA knockdown is demonstrated in Fig. 3B.

As is the case with Skp1 knockdown (Fig. 1), depletion of FBXW11 impaired the replication of WT RVFV, as measured by the reduction of the RVFV N signal. To clarify whether this is again due to the partial stabilization and activation of PKR, we performed infection and knockdown experiments with PKR-deficient cells. As shown in Fig. 3C, knockdown of FBXW11 in PKR-expressing cells lowered WT RVFV titers by a factor of 5, while no such difference was observed in PKR-deficient cells. Moreover, NSs mutant clone 13 shows no PKR-dependent titer reduction in FBXW11 knockdown cells.

These data indicate that the degradation of PKR by RVFV NSs is partially mediated by the E3 ubiquitin ligase component FBXW11 and that the virus requires this host factor for optimal replication to counteract the protein synthesis shutoff caused by PKR.

### FBXW11 acts in concert with the E3 ligase β-TRCP1.

**FIG 4:**
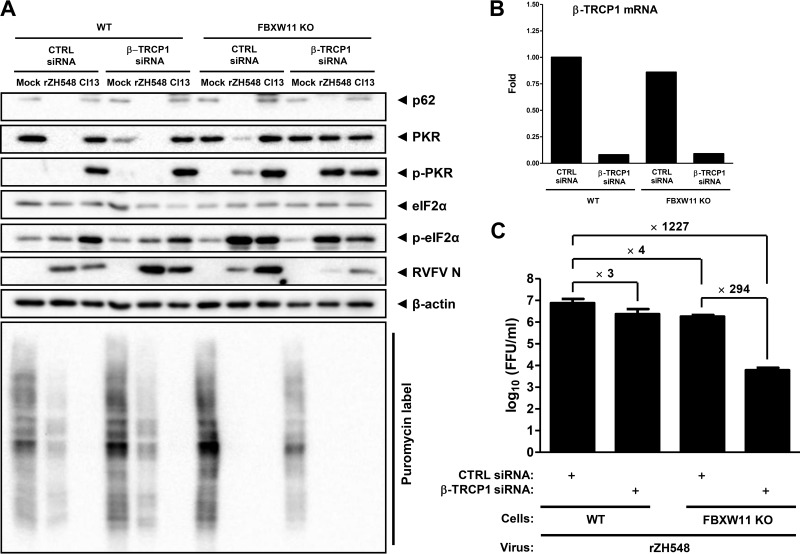
Involvement of β-TRCP1 in PKR degradation by NSs. (A) WT human A549 cells or A549 cells with a CRISPR/Cas9-mediated knockout of the FBXW11 gene (FBXW11 KO) were transfected with siRNAs against β-TRCP1 mRNA and then infected with WT RVFV (rZH548) or clone 13 (Cl13) at an MOI of 10 for 6 h. Nascent protein chains were labeled by incubating cells with puromycin for 30 min before lysis. Samples were analyzed by Western blotting for the presence of the proteins indicated and for the puromycin label. (B) Efficiency of the β-TRCP1 siRNA knockdown, shown by RT-quantitative PCR. (C) FBXW11- and β-TRCP1-dependent growth of RVFV. A549 WT and FBXW11 knockout cells were treated with siRNAs against β-TRCP1 or with a control (CTRL) siRNA and infected with rZH548 at an MOI of 1, and virus titers were determined at 16 h p.i. Three replicate wells were infected per experiment. The experiment was repeated three times, and mean values and standard deviations of the three independent repeats, as well as fold differences between siRNA treatments, are shown.

Although a contribution of FBXW11 to NSs-mediated PKR degradation is obvious from the experiments presented here, depletion of FBXW11 did not entirely rescue PKR levels. This is in contrast to the results obtained by knockdown of the general SCF complex component Skp1, which protected PKR levels from the action of NSs much better (Fig. 1). We therefore considered that an additional F-box protein may cooperate with FBXW11 to impair PKR in infected cells. Interestingly, there exists an F-box protein that has the same substrate selectivity as FBXW11 and is structurally similar to it, i.e., FBXW1, which is better known as β-TRCP1 (33). To test the potential involvement of β-TRCP1 in addition to FBXW11, we generated an FBXW11 knockout cell line by CRISPR/Cas9 technology (see Materials and Methods). This cell line recapitulated the WT RVFV phenotype expected from the previous FBXW11 siRNA experiments, namely, partial rescue of PKR but complete degradation of TFIIH-p62, as well as slight PKR phosphorylation, strong eIF2α phosphorylation, and shutoff of protein synthesis at 6 h p.i. (Fig. 4A). Strikingly, when the FBXW11 knockout cells were transfected with an siRNA against β-TRCP1 (Fig. 4B), PKR was entirely protected from NSs-mediated degradation (see Fig. 4A). Consequently, virus infection was reduced even more than in cells depleted only of FBXW11 (Fig. 4C). The full phosphorylation of PKR and the complete degradation of TFIIH-p62 demonstrate that the degree of virus infection is nonetheless sufficient to fully activate host responses and degrade other target proteins. Depletion of just β-TRCP1 from WT cells, in contrast, had no apparent bearing on PKR degradation by NSs, albeit some titer reduction was observed (see Fig. 4C). We therefore conclude that RVFV NSs requires both F-box proteins, FBXW11 (mainly) and β-TRCP1 (as a helper to remove residual PKR), to entirely destroy antiviral PKR in infected cells.

**FIG 5:**
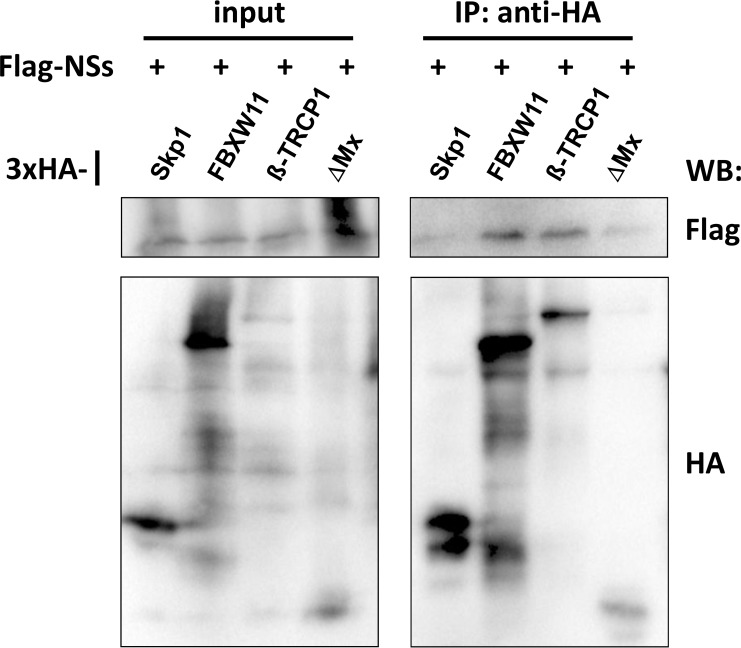
Interaction of NSs with FBXW11 and β-TRCP1. Coimmunoprecipitation assay. *In vitro*-translated, 3×HA-tagged Skp1, FBXW11, β-TRCP1, and ΔMx were coupled to magnetic beads; coincubated with *in vitro*-translated, 3×FLAG-tagged RVFV NSs; and precipitated. Input proteins (left panel) and immunoprecipitates (IP; right panel) were analyzed by Western blotting (WB).

We had previously shown that the degradation of TFIIH-p62 is based on an interaction of RVFV NSs with FBXO3 (22). In order to elucidate whether degradation of PKR occurs by a similar mechanism, we performed coimmunoprecipitation assays. FBXW11 and β-TRCP1 were expressed *in vitro*, coupled to magnetic beads via an N-terminal 3×HA epitope tag, and then coincubated with 3×FLAG-tagged RVFV NSs. In addition, 3×HA-tagged Skp1, the universal cofactor and interactor of F-box proteins, and an N-terminal fragment of the human MxA protein (ΔMx; used as a negative control) were included in the analysis. The Western blot assay of the input control shows expression for all interaction candidates and controls (Fig. 5, left panel). The immunoprecipitates showed equal immunoprecipitation levels of the 3×HA-tagged bait proteins. RVFV NSs, however, coimmunoprecipitated only with FBXW11 and β-TRCP1 and not with Skp1 or the negative control, ΔMx (Fig. 5, right panel). These results are in line with the siRNA experiments (see Fig. 4) and indicate that RVFV NSs interacts with these F-box proteins to destroy PKR.

## DISCUSSION

The nonstructural protein NSs is the major virulence factor of RVFV. It acts by multiple, apparently independent, strategies. On the one hand, NSs suppresses antiviral gene expression by engaging the transcriptional repressor SAP30 and by sequestering and mediating the degradation of subunits of the general transcription factor TFIIH (19, 21–23). On the other hand, NSs destroys the antiviral kinase PKR (9, 10). The destruction of both TFIIH subunit p62 and PKR by NSs occurs via the proteasomal pathway. We previously showed that for degradation of TFIIH-p62, NSs interacts with the E3 ubiquitin ligase subunit FBXO3 (22). Here, we demonstrate that for PKR degradation, the two E3 ubiquitin ligases FBXW11 and β-TRCP1 are recruited. Of note, when our manuscript was under review, Mudhasani et al. reported similar findings (34).

FBXO3, FBXW11, and β-TRCP1 are substrate recognition subunits of the modular SCF-type E3 ubiquitin ligase complexes. Consequently, the conserved constituent of SCF complexes, Skp1, is involved in the degradation of both TFIIH-p62 and PKR. Thus, SCF-type ubiquitin ligases are key determinants of the virulence of RVFV, as NSs employs different SCF modules to target centrally important host factors of the antiviral response.

Interference with the NSs/SCF network could be a promising therapeutic strategy against Rift Valley fever. In fact, an inhibitor of FBXO3 is available (35). Although the depletion of FBXO3 by siRNA does not impact RVFV replication in cell culture, it rescues the induction of antiviral cytokines like IFN-β and IP-10 by more than 1 order of magnitude (22). At first glance, Skp1, FBXW11, and β-TRCP1 appear to be most suitable targets for therapy, as depletion of these factors has a major impact on viral replication. It should be noted, however, that Skp1 is a conserved element of many SCF ligases that is essential for cellular integrity and function (30, 32, 33). Similarly, FBXW11 and β-TRCP1 are important players in innate immunity, as they degrade IκB to activate the master immune regulator NF-κB and regulate many reactions important for stress responses, immune signaling, the cell cycle, and DNA damage responses, to name a few (33). Thus, broad inhibition of Skp1, FBXW11, or β-TRCP1 would almost certainly have serious side effects.

Only NSs-expressing WT RVFV, but not NSs-deficient clone 13, was prone to the strong, PKR-dependent antiviral effect of FBXW11 knockdown. This indicates that an NSs that cannot fulfill one of its major functions—PKR degradation—becomes a burden for the virus. Skp1, in contrast, is required for both WT RVFV and clone 13. The biggest effect of Skp1 knockdown was nonetheless on WT RVFV in the presence of PKR, but even in the absence of NSs and/or PKR, there remained a certain dependence of viral replication on Skp1. Thus, while an FBXW11-containing E3 ubiquitin ligase is specifically required for PKR degradation, other E3 ubiquitin ligases of the SCF type may play a general role in RVFV replication, perhaps explaining the inhibitory effect of proteasomal inhibitors on RVFV replication (9, 22, 23).

To maximally downregulate PKR levels, NSs required both β-TRCP1 and FBXW11. There seems to be a certain hierarchy, because β-TRCP1 depletion alone did not show any effect on PKR levels. Apparently, β-TRCP1 can act as a backup for the principal PKR destroyer FBXW11. The mechanism of this dual engagement of highly related E3 ligases remains to be investigated but is most likely based on the high structural similarity of FBXW11 and β-TRCP1 (36, 37). The key role of FBXW11/β-TRCP1 in NF-κB signaling may imply a contribution of these factors to the pathogenicity of RVFV that is beyond the NSs-mediated PKR degradation described here.

In summary, we have identified the host cell factors that are required by RVFV to overcome the major antiviral IFN effector PKR. The strong dependency of RVFV replication on a functional PKR destruction cascade raises hope for the development of specific antiviral therapeutics.
